# High binding affinity of repressor IolR avoids costs of untimely induction of *myo*-inositol utilization by *Salmonella* Typhimurium

**DOI:** 10.1038/srep44362

**Published:** 2017-03-14

**Authors:** Jessica Hellinckx, Ralf Heermann, Angela Felsl, Thilo M. Fuchs

**Affiliations:** 1 Lehrstuhl für Mikrobielle Ökologie, Zentralinstitut für Ernährungs- und Lebensmittelforschung ZIEL, Technische Universität München, Weihenstephaner Berg 3, 85354 Freising, Germany; 2 Biozentrum, Bereich Mikrobiologie, Ludwig-Maximilians-Universität München, Großhaderner Str. 2–4, 82152 Martinsried/München, Germany; 3 Friedrich-Loeffler-Institut, Institut für Molekulare Pathogenese, Naumburger Str. 96a, 07743 Jena, Germany

## Abstract

Growth of *Salmonella enterica* serovar Typhimurium strain 14028 with *myo*-inositol (MI) is characterized by a bistable phenotype that manifests with an extraordinarily long (34 h) and variable lag phase. When cells were pre-grown in minimal medium with MI, however, the lag phase shortened drastically to eight hours, and to six hours in the absence of the regulator IolR. To unravel the molecular mechanism behind this phenomenon, we investigated this repressor in more detail. Flow cytometry analysis of the *iolR* promoter at a single cell level demonstrated bistability of its transcriptional activation. Electrophoretic mobility shift assays were used to narrow the potential binding region of IolR and identified at least two binding sites in most *iol* gene promoters. Surface plasmon resonance spectroscopy quantified IolR binding and indicated its putative oligomerization and high binding affinity towards specific *iol* gene promoters. In competitive assays, the *iolR* deletion mutant, in which *iol* gene repression is abolished, showed a severe growth disadvantage of ~15% relative to the parental strain in rich medium. We hypothesize that the strong repression of *iol* gene transcription is required to maintain a balance between metabolic flexibility and fitness costs, which follow the inopportune induction of an unusual metabolic pathway.

Bacterial pathogens often encounter limited availability of carbon, nitrogen and energy sources during their life cycles due to competition with the host organism and the commensal microbiota for nutrients. Increasing evidence suggests that enteric pathogens have developed specific metabolic strategies to overcome those restrictions and thus increase their fitness in the environment and *in vivo*. Metabolic adaptations of salmonellae during infection include the degradation of ethanolamine cleaved from phospholipids, fucose derived from mucosal glycoconjugates, 1,2-propanediol, the fermentation product of fucose or rhamnose^1^, and other potential substrates not accessible to commensal bacteria or present in competition-free microenvironments.

The ability of *Salmonella enterica* serovar Typhimurium (*S.* Typhimurium) to grow with *myo*-inositol (MI) as its sole carbon and energy source provides another example of an adaptive metabolic pathway with a potential role *in vivo*. In *S.* Typhimurium 14028, the *iol* genes responsible for MI utilization are located on a 22.6-kb genomic island (GEI4417/4436)^2^. The catabolic pathway requires the IolG- and IolE-mediated convertion of MI to 3D-trihydroxycyclohexane-1,2-dione, which is further hydrolysed by IolD. Next, 5-deoxy-glucuronic acid is isomerized by IolB to 2-deoxy-5-keto-D-gluconic acid, which is in turn phosphorylated by the kinase IolC and degraded to dihydroxyacetone phosphate, acetyl coenzyme A and CO_2_. The activator ReiD has been shown to interact with the promoter of *iolE* and thus to positively regulate *iol* gene expression^3^.

A remarkable and unique property of *S.* Typhimurium 14028 cultivated in minimal medium (MM) with MI is the manifestation of a heterogeneous growth phenotype that is abolished in the absence of the *iol* gene repressor IolR or the presence of at least 0.55% CO_2_^4^. This phenotypic heterogeneity correlates with the bistable expression of *iolE* and *iolG*, the products of which catalyze the first steps in MI degradation. At the single cell level, fluorescence microscopy and flow cytometry (FC) analyses revealed that increasing numbers of cells exhibit P_*iolE*_ activation during growth with MI^4^. More recently, we demonstrated that a shift from MI medium to LB medium is characterized by a “memory” of salmonellae for the former medium, and that this hysteresis is successively lost after 8 hours in a medium without MI^5^.

A key player in MI utilization is the negative autoregulator IolR that represses in rich medium all but one promoter of the *iol* genes involved in MI degradation, including its own promoter^2^ and that of the *iolT1* gene encoding the predominant MI transporter in *S.* Typhimurium 14028^6^. An intermediate of MI degradation, 2-deoxy-5-keto-D-gluconic acid 6-phosphate (DKGP), has been shown to antagonize IolR binding, thus inducing the expression of *iol* genes^7^,^8^. Here, we investigated IolR in greater detail, as this repressor plays a pivotal role in the coordinated control of MI degradation and the observed phenotypic heterogeneity. Using quantitative growth data of the wildtype and the *iolR* deletion mutant, the key parameters of growth with MI were delineated. The transcriptional activity of the *iolR* promoter was analyzed at a single cell level via FC. Electrophoretic mobility shift assays (EMSAs) and surface plasmon resonance (SPR) spectroscopy qualitatively characterized the binding properties of IolR towards its target promoters. Finally, competitive growth assays suggested that induced cells suffer a growth disadvantage in the absence of MI, a constraint that might contribute to the tight repression of *iol* gene transcription in rich medium.

## Results

### IolR contributes to lag-phase length during growth with MI

**Figure 1:**
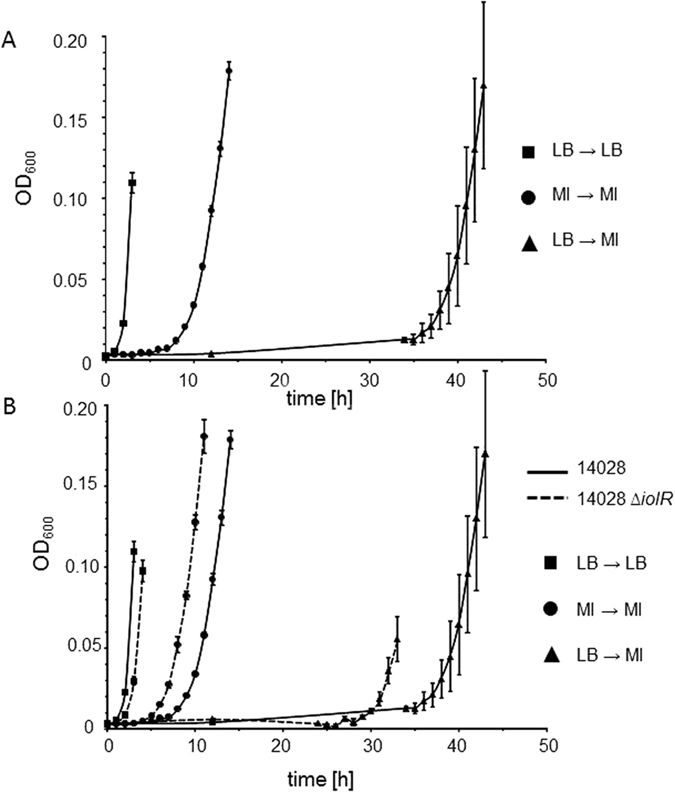
Lag phases of *S.* Typhimurium during growth with MI. (**A**) Strain 14028 pre-grown in MM/MI and then diluted 1:500 into MM/MI exhibited a much shorter lag phase in comparison with that of a culture inoculated with a pre-culture in LB medium. The growth curve of a culture taken from LB medium and re-inoculated into the same medium is shown as a control. (**B**) Lag phases were shortened by deletion of the repressor gene *iolR*. Growth curves of 14028 from (**A**) are displayed in black, and those of 14028 ∆*iolR* are indicated by dashed lines. Growth curves were derived as described in (**A**). Standard deviations were calculated from three biologically independent measurements.

The lag phase of *S*. Typhimurium in the presence of MI has not previously been evaluated in detail. Therefore, we measured cultures that were inoculated with cells pregrown either in LB medium or on solid MM/MI. An overnight culture of 14028 in LB medium was adjusted to an OD_600_ = 0.8 and then diluted 1:500 into MM/MI. When this dilution was further cultivated under standard conditions, a lag phase of approximately 34 h was observed (Fig. 1A). The length of this lag phase was characterized by a low level reproducibility, which was reflective of the stochastic and heterogeneous growth behaviours under this condition as indicated by high standard deviations^4^. In contrast, when strain 14028 was pregrown on MM/MI agar plates, adjusted to an OD_600_ = 0.8 and diluted as described, the resulting culture exhibited a much shorter lag phase of 8 h, which could be attributed to the fact that the cells had already adapted to MI utilization. As a control, a growth curve of strain 14028 pregrown in LB medium that was subsequently diluted into fresh LB medium showed a lag phase of <2 h (Fig. 1A). The standard deviations of data from the experiment using 14028 pregrown on MM/MI were similar to those observed in the control, indicating that strain 14028 grows homogeneously in MM after preadaptation to this medium.

Next, mutant 14028 ∆*iolR*, which lacks the repressor IolR, was investigated under the experimental settings described above. When an LB culture was used to inoculate a MM/MI culture, we observed a reduction in the lag phase to 30 h, as well as a reduced population heterogeneity, as indicated by the low standard deviation, relative to the growth of the parental strain (Fig. 1B). When the mutant was pre-grown on MM/MI prior to the dilution into fresh MM/MI, a lag phase of 6 h was observed compared with 8 h observed for strain 14028 under the same conditions. In the LB control experiment, the lag phase of the ∆*iolR* mutant was one hour longer than that of strain 14028, possibly because the targeted induction of the MI degradation pathway in 14028 Δ*iolR* led to weak growth attenuation (see below).

Taken together, the lag phase of *S.* Typhimurium growth in MM/MI ranges between 6 h and 34 h under the conditions used herein.

### Temporal analysis of P_*iolR*_ transcription by FC

**Figure 2:**
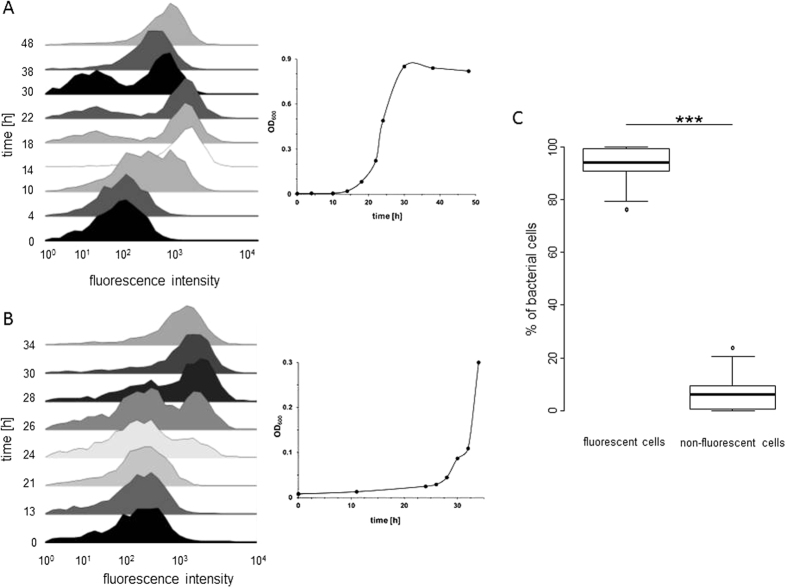
Temporal FC analysis of P_*iolR*_ activity in MM with MI. (**A**) MvP101 P_*iolR*_::*gfp* or (**B**) MvP101 *iolR*::*gfp* grown in LB medium was adjusted to an OD_600_ of 0.8, diluted 1:500 into MM/MI and incubated at 37 °C without agitation. At the indicated time points during growth, samples were collected and GFP-expressing cells were quantified by FC. The abscissa of each histogram represents the green fluorescence intensity at 515–545 nm on a bi-exponential scale, and the ordinate represents the numbers of bacteria relative to the maximal cell counts. Each histogram shows one representative data set of three biologically independent measurements. The inset illustrates the growth phase of the population during sample acquisition. (**C**) FC measurement of MvP101 P_*rpsM*_::*gfp* as a control. The percentages of *gfp*-expressing cells (left) and non-fluorescent cells (right) are indicated. Samples were collected during a 48-h growth period after inoculation, and the average values and standard deviations of three independent growth experiments are shown; ***p < 0.001.

We have previously demonstrated at the single cell level that the promoter of *iolE* is bistable^4^. The promoter of *iolR*, P_*iolR*_, is transcriptionally active in the presence of both glucose and MI^2^. To investigate the transcription of *iolR* in more detail, we performed a temporal FC analysis of strain MvP101 with the transcriptional fusion P_*iolR*_::*gfp* during growth in MM with MI. Figure 2A shows that all cells exhibit fluorescence below the threshold level at time point zero (i. e. when LB-cultured cells were inoculated into MM/MI medium). During cultivation until the end of the lag phase, the number of cells exhibiting a higher level of fluorescence steadily increased, and two subpopulations with a low and a high expression level were detected. 14 hours after inoculation, a temporary increase of the fluorescence activity was visible. At this time point, the division rate of the cells was still low, possibly resulting in an accumulation of GFP within the cells. To test whether the translation of *iolR* is also subject to bistability, we measured the fluorescence of strain MvP101 harboring the translational fusion *iolR*::*gfp* in the presence of MI. The data shown in Fig. 2B generally resemble those obtained with a transcriptional reporter fusion protein, indicating that the transcriptional bistability is not abolished during IolR production by strain MvP101. Samples of the control strain MvP101 P_*rpsM*_::*gfp* with constitutive expression were collected and measured for up to 48 h of growth in MM/MI, during which time we observed averages of approximately 93% cells with and 7% cells without *gfp* expression (Fig. 2C). As the fusion P_*rpsM*_::*gfp* is expected to be active in viable cells only, the data demonstrate that the majority of cells without significant fluorescence activity are not dead cells.

As an indicator for heterogeneous gene expression within a population, we calculated the variability in the fluorescence intensity, or the noise-level^9^, over all the growth phases in MM/MI of the two reporter strains described above (Table 1). The noise-levels of both MvP101 P_*iolR*_::*gfp* and MvP101 *iolR*::*gfp* were highest (1.67 ± 0.13) at the beginning of the lag phase and decreased to minimum levels (0.08 ± 0.0) during the stationary phase, when the cell populations showed more homogeneous gene expression. In contrast, the control strain MvP101 P_*rpsM*_::*gfp* exhibited an average noise-level of 0.01 ± 0.01 during all growth stages.

To summarize, these findings demonstrate a bistable transcriptional activity of P_*iolR*_ similar to that of P_*iolE*_.

### Interaction of IolR-His_6_ with *iol* gene promoters

**Figure 3:**
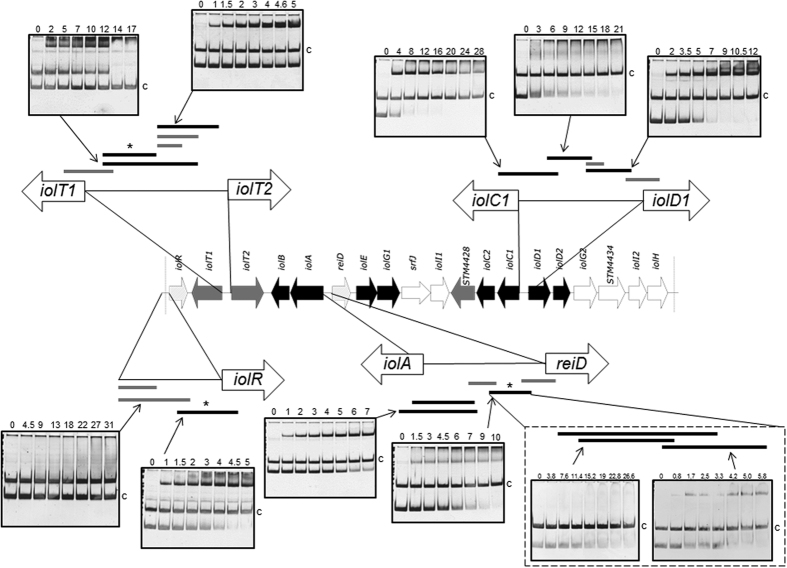
IolR binding to promoter regions within GEI4417/4436. The genomic regions of the island are highlighted, and the different fragments used for EMSAs are shown. Black fragments were bound by IolR, grey fragments not. The molar excess of protein over DNA is indicated above the gels. The *argS* (100 ng) promoter fragment of served as a negative control and competitor (c) DNA, and the first lane in each EMSA was loaded without IolR. Asterisks indicate fragments selected for SPR spectroscopy.

The binding of IolR to most *iol* promotor regions was demonstrated previously^2^,^3^,^6^. To delineate a molecular model for the interaction of IolR and its target sites, we here performed EMSAs using different-length promoter fragments that represented possible IolR binding regions. The regulator was overproduced in *E. coli* BL21(DE3) and purified as described in the Methods. Putative promoter fragments of *iolR, iolT1, iolT2, iolA, reiD, iolC1* and *iolD1* were incubated without or with increasing amounts of purified IolR-His_6_ protein, and the resulting protein-DNA complexes were separated on a polyacrylamide (PAA) gel^10^. The EMSAs demonstrated the specificity of IolR-His_6_ to its target DNA and narrowed the binding region of IolR within the promoters P_*iolR*_, P_*iolT1*_, P_*iolT2*_, P_*iolA*_, P_*reiD*_, P_*iolC1*_ and P_*iolD1*_ to a length of approximately 100 bp (Fig. 3). The molecular excess of protein over DNA for quantitative binding of most promoters tested was in a range between 5 to 21 and thus very low in comparison with other repressors tested under similar conditions^11^,^12^, pointing to a strong affinity of IolR to its target sequences. The bandshift experiments with the promoters P_*iolT1*_, P_*iolD1*_, P_*iolR*_, P_*iolC1*_ and P_*reiD*_ exhibited at least two retarded protein/DNA complexes, suggesting an oligomerization of IolR with increasing protein concentration. We then investigated two fragments of 74 and 81 bp from P_*reiD*_ with an overlap of only 7 nucleotides by EMSA and observed a binding of IolR-His_6_ to both sequences, indicating the presence of two independent IolR target sites (Fig. 3). Moreover, the molar excesses of approximately 20 and 5, respectively, for a complete shift of these two P_*reiD*_ fragments demonstrate different affinities of IolR to the two binding sites.

### Quantification of DNA-binding by IolR-His_6_

**Figure 4:**
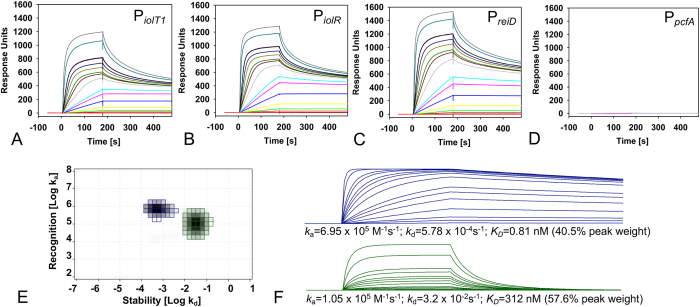
SPR spectroscopy of IolR binding to P_*iolT1*_, P_*iolR*_ and P_*reiD*_. The biotin-labeled DNA fragments P_*iolT1*_ (**A**), P_*iolR*_ (**B**), and P_*reiD*_ (**C**) and control fragment P_*pcfA*_ (**D**) were captured on a streptavidin-coated sensor chip, and purified IolR was passed over the chip at a flow rate of 30 μl/min and temperature of 25 °C [concentrations of 0, 0.066, 0.165, 0.33, 0.66, 1.65, 3.3, 4.95, 6.6, 13.2, 19.8, 26.4, 33, 49.5, 66 (black and purple lines, internal reference), 165 and 330 nM] using a contact (association) time of 180 sec, followed by a 300-sec dissociation phase. The increase in RU correlates with an increasing IolR concentration. (**E**) IM analysis of the IolR-P_*iolT1*_ interaction. The green and blue spots represent both interactions of IolR with the P_*iolR*_ DNA. The separate sensorgrams with the specific K_D_ values calculated from ON/OFF-rates map are shown in (**F**), together with the quantification of the *in silico* binding kinetics, e. g. the calculated association (k_a_) and dissociation (k_d_) rates as well as the quantitative portion of the total peak.

To analyse the interaction of IolR with its target sites identified above in more detail, we performed a kinetic analysis of IolR-His_6_ binding to P_*iolT1*_, P_*iolR*_ and P_*reiD*_ via SPR spectroscopy using fragments selected from the EMSAs. IolR was confirmed to specifically interact with P_*iolT1*_, P_*iolR*_ and P_*reiD*_, whereas no interaction was detected with the control promoter P_*pcfA*_ (Fig. 4A–D). Because the maximal binding capacity (*R*_max_) for each sensorgram is >1.000 responsive units (RU) and the *R*_max_ for an 1:1 interaction is expected to be approximately 20 RU, these data suggest that IolR binds as a higher oligomer and/or that multiple binding sites for IolR exist within the *iol* promoter regions. The finding that none of the sensorgrams followed 1:1 binding kinetics, as indicated by the hyperbolic curve shape, underscores the assumption that the sensorgrams represent the sums of different binding events. To calculate reliable binding constants and kinetic parameters, we performed an IM analysis and determined the individual binding events for the SPR spectroscopy curves of IolR-P_*iolT1*_. Based on the sensorgrams, the IM analysis identified two clearly separated peak values (Fig. 4E) that represented a high-affinity interaction, and an interaction with an approximately 400-fold lower affinity. The first peak (blue) had a peak weight of 40.5%, an ON-rate of 6.95 × 10^5^ M^−1^s^−1^ and an OFF-rate of 5.78 × 10^−4^s^−1^, resulting in an overall high affinity of 0.81 nM (Fig. 4E and F, upper panel). The second peak (green) had a peak weight of 57.6% and an ON-rate of 1.05 × 10^5^ M^−1^s^−1^. The OFF-rate for this interaction was determined to be 3.2 × 10^−2^s^−1^, which was 50-fold higher than the first peak (depicted in blue), corresponding to a lower *K*_*D*_ of 312 nM (Fig. 4E and F, lower panel). We therefore conclude that two IolR binding sites with different affinities exist in the P_*iolT1*_ promoter region, which are occupied by a IolR oligomer that influences target gene expression. Because each binding peak accounts for approximately 50%, it is hypothesized that both DNA-binding sites are bound by equal numbers of IolR molecules.

According to the OneToTwo evaluation algorithm, the binding kinetics underlying the sensorgrams of IolR-P_*iolR*_ and IolR-P_*reiD*_ interactions were nearly identical to that of the IolR-P_*iolT1*_ complex. The ON-rates for the IolR-P_*iolR*_ and IolR-P_*reiD*_ interaction were determined to be k_a1_ = 1.3 × 10^5^ M^−1^s^−1^ and k_a2_ = 2.4 × 10^5^ M^−1^s^−1^, and k_a1_ = 1.5 × 10^5^ M^−1^s^−1^ and k_a2_ = 2.1 × 10^5^ M^−1^s^−1^, respectively. The corresponding OFF-rates were k_d1_ = 5.5 × 10^−2^ s^−1^ and k_d2_ = 1.4 × 10^−3^ s^−1^ and of k_d1_ = 4.9 × 10^−2^/s and k_d2_ = 1.4 × 10^−3^/s, respectively. For both promoters, these data yielded to two binding events that differed with respect to the overall affinity (K_D 1_ = 425 nM; K_D2_ = 6.0 nM, and K_D1_ = 321 nM; K_D2_ = 6.6 nM, respectively).

Taken together, IolR binds at two sites within the respective *iol* gene promoter region, with one site bound with very high affinity in the low nanomolar range, and one site with lower affinity. The binding kinetics of the higher affinity-site with a high ON-rate and a very slow OFF-rate are comparable to those of antigen-antibody interactions^13^,^14^, thus demonstrating a remarkable high affinity of IolR to the promoter regions of *iolT1, iolR*, and *reiD*.

### Fitness of cells with constitutive *iol* gene expression

**Figure 5:**
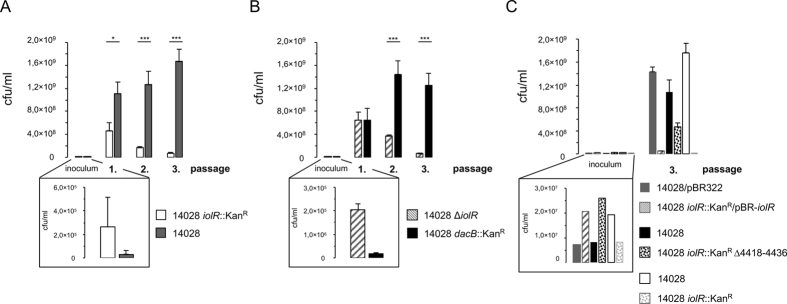
Competitive fitness assays of 14028 strains versus their *iolR* mutants. Strains were grown separately in LB medium to an OD_600_ of 0.5 and mixed together in a ratio of approximately 1:10 for 14028 vs. *iolR*::Kan^R^ (**A**) and 14028 *dacB*::Kan^R^ vs. ∆*iolR* (**B**). This mixture was used to inoculate 50 ml LB medium in an appropriate dilution of 1:250 to 1:1000. After a 24-h incubation, the cfu/ml were determined by plating culture samples on LB medium with or without kanamycin, and an aliquot of each culture was diluted 1:500 into fresh LB medium. This step was repeated twice. For each passage, the percentages of strains within the cultivated mixture are shown. (**C**) Competitive growth experiment with complemented strain 14028 *iolR*::kan^R^/pBR-*iolR* against 14028/pBR322, and with strains 14028 and 14028 *iolR*::Kan^R^ Δ4418–4436; 14028 and 14028 *iolR*::Kan^R^ were used as a control. The inoculum was diluted 1:500. Cell numbers were calculated only after the third passage. Standard deviations were calculated from triplicate measurements of three independent experiments. Significance values were below 1% (p < 0.01*) or below 0.01% (p < 0,0001***).

The high binding affinity of IolR prompted us to speculate that in media lacking MI, cells with an activated MI utilization pathway would exhibit attenuated growth compared to those with silenced *iol* genes. As demonstrated previously through a promoter probe and FC analysis^2^,^4^, deletion of *iolR* for results in constitutive *iol* gene transcription regardless of the growth conditions. Therefore, we investigated the relative growth fitness of strain 14028 *iolR*::Kan^R^ in comparison with the kanamycin-sensitive (Kan^S^) strain 14028 in a competitive growth experiment. Both strains were grown separately in LB medium to an OD_600_ of 0.5 and subsequently mixed in a 1:10 ratio (14028:14028 *iolR*::Kan^R^). To control the ratio of the two strains, aliquots were plated to determine the numbers of colony forming units (cfu)/ml. The mixture (“inoculum”) was appropriately diluted into LB medium. After incubation for 24 h without agitation, the numbers of viable Kan^R^ and Kan^S^ cells were determined by plating dilutions of the culture on solid LB medium with or without kanamycin. To determine the longer-term strain fitness, 0.1 ml of the 24-h culture was inoculated 1:500 into fresh LB medium and incubated for 1 day. Then, the cfu/ml of each strain was determined and an aliquot of this culture was used to inoculate fresh medium. This third culture was also incubated for 24 h and analyzed as described above. As a measure of the relative fitness of each strain, we calculated the cfu/ml of each strain after each of the three passages. The experiments started with a roughly 10-fold excess of strain 14028 *iolR*::Kan^R^ versus 14028 (89.6%, ±0.62%). Then, the percentage of the *iolR*-negative strain in the mixture decreased drastically to 29.2% (±7.46%) after the first passage, to 11.8% (±2.36%) after the second passage and to 4.2% (±0.51%) after the third passage (Fig. 5A), indicating a significant reduction in the fitness of strain 14028 *iolR*::Kan^R^ relative to strain 14028.

To exclude the possibility that the decrease of the *iolR* mutant fitness was related to the energetic cost of the Kan^R^ cassette, we generated a mutant (14028 *dacB*::Kan^R^) in which *dacB* was replaced with a Kan^R^ cassette. *DacB* encodes penicillin binding protein 5, which exhibits DD-carboxypeptidase activity, and does not affect *S*. Typhimurium growth^15^. Using the kanamycin-sensitive, non-polar deletion mutant 14028 ∆*iolR* as a competitor strain, we repeated the described experiment using a strain ratio as mentioned above. Again, we observed a strong growth retardation of the *iolR* mutant compared to strain 14028 *dacB*::Kan^R^, in which *iol* gene transcription was silenced by IolR (Fig. 5B). The initial percentage of strain 14028 ∆*iolR* in the mixture was 91.5% (±1.04%) and decreased to 49.7% (±6.25%), 19.9% (±7.16%) and 4.6% (±0.73%) after the first, second and third passages, respectively. To quantify the fitness cost, we compared the division rates of 14028 (0.39 h^−1^) and 14028 *iolR*::Kan^R^ (0.33 h^−1^) during the third passage and calculated a 15.4% reduction of the growth rate.

We then tested 14028 *iolR*::Kan^R^/pBR-*iolR* against 14028/pBR322 in a similar manner. The complementation of *iolR* strongly reduced the growth advantage of strain 14028 over the mutant 14028 *iolR*::Kan^R^ (initial ratio 2.4:1 versus 441:1 after three passages) to a ratio of 29:1 (Fig. 5C). This data refute the hypothesis that incidental mutations outside the *iolR* gene played a role to the growth phenotype observed above.

Recently, IolR was reported to activate the expression of genes encoding components of the reversible lysine acetylation system^16^. To therefore exclude the possibility that any function beside *iol* gene repression contributes to the growth attenuation of 14028 *iolR*::Kan^R^, we performed a competitive growth experiment with strains 14028 and 14028 *iolR*::Kan^R^ Δ4418–4436. Here, the initial ratio of 1:3 between the strains shifted to a ratio of 2.3:1 after three passages (Fig. 5C) in contrast to the control experiment with strains 14028 and 14028 *iolR*::Kan^R^ (ratio 2.4:1 versus 441:1), demonstrating that the manifestation of fitness costs of a *iolR* mutant requires the presence of all genes of the genomic island. As an additional control, the growth of each strain in LB medium was monitored to exclude non-specific growth deficiencies, but no differences in growth were observed (Figure S1).

Taken together, both 14028 ∆*iolR* and 14028 *iolR*::Kan^R^, which feature artificially arrested *iol* gene expression, exhibited a fitness disadvantage in rich medium in comparison with strains 14028 *dacB*::Kan^R^ or 14028, in which the *iol* genes are tightly repressed. We conclude that tight repression of the MI utilization pathway in *S*. Typhimurium is likely a consequence of the fitness costs of inappropriately relaxed *iol* genes, resulting in a constraint of the number of cells with activated *iol* genes within an isogenic population.

## Discussion

Recent findings suggested that the MI utilisation pathway of *S.* Typhimurium is very tightly repressed in the absence of MI, and that this repression is mainly achieved by the repressor IolR^2^. This explanation is biologically reasonable as *S.* Typhimurium can utilize a number of carbon sources, among which MI is not the most preferable^1^. Strong *iol* gene repression is phenotypically manifested by the start of logarithmic growth of cells that were pre-grown in LB medium diluted 1:500 into MM with MI after only 35 h in culture. In contrast, a much shorter lag phase similar to that observed in rich medium was obtained when *Salmonella* cells had been pre-grown in MI-containing MM and were thus adapted to the MI-utilisation metabolic pathway. Under this experimental condition the bistable phenotype was abolished, as indicated by homogeneous growth behavior^4^ and low variability in the lag phase duration, which resembled that observed in rich medium. We therefore investigated the repressor IolR here in more detail.

The SPR spectroscopy performed in this study demonstrated high binding affinities of IolR to the promoters P_*iolT1*_ and P_*reiD*_, which control the genes encoding the main MI transporter and the activator of the MI degradation cascade. Additionally, this study revealed the oligomerization of IolR as indicated by the retarded protein/DNA complexes (Fig. 3). IolR oligomerization was also suggested by SPR spectroscopy. Calculating the max binding capacity of the chip surface at a simple one-to one interaction would result in an RU value of ~20. Since a maximal RU of 1200 RU was measured, we conclude that IolR binds as a higher oligomer even if two binding sites are present in a promoter fragment. Analysis of the SPR sensorgrams then identified one high ON/high OFF and one high ON/slow OFF binding sites of IolR within P_*iolT1*_, P_*iolR*_ and P_*reiD*_ with different affinities. The detection of these two binding sites with high-affinity (A-site) and low-affinity (B-site) suggests a complex, cooperative DNA-binding mechanism of IolR similar to the so-called AB-BA mechanism that was recently described for the bacterial response regulator YpdB^17^. In this example, subsequent and cooperative promoter binding by several YpdB copies and a rapid successive promotor clearance results in a pulse-like gene expression.

**Figure 6:**
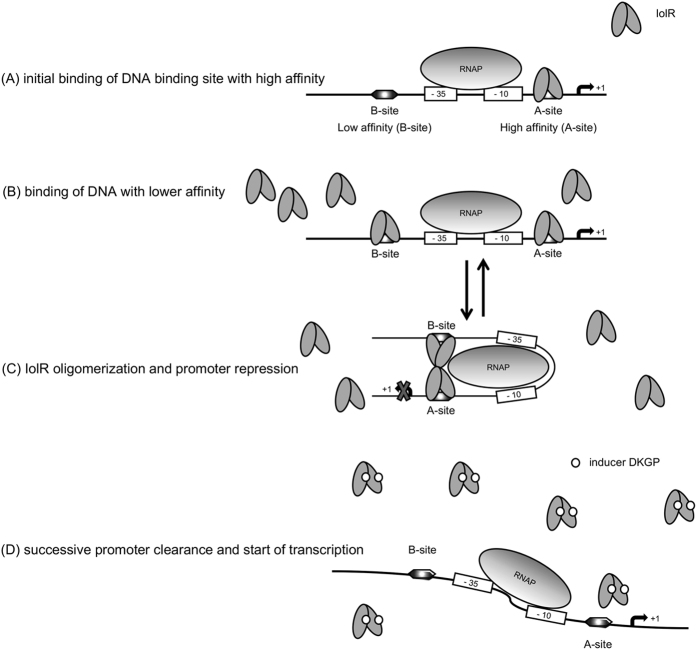
Model of IolR interaction with its promoters. Two different binding sites for IolR with high-affinity (A-site) and low-affinity (B-site) are present in the *iol* promoters exemplified here by P_*reiD*_. (**A**) Initial binding of IolR dimers to the A-site of the promoter hampering RNA polymerase (RNAP) activity. (**B**) In case of higher copy numbers of IolR, also the B-site with lower affinity will be occupied by the repressor. (**C**) Oligomerization of IolR dimers result in trapping of the RNAP and completely inhibits *reiD* transcription. (**D**) Binding of the putative inducer (ligand) DKGP abolishes IolR binding, allowing the RNAP to start transcription. In this model, the binding or release of the B-site by IolR depends on the IolR copy numbers and the concentration of the inducer, leading to a pulse-like and highly reversible gene expression; together with the bistable autoregulation of *iolR*, the equilibrium between status (**B**) and (**C**) is decisive for *iol* gene transcription.

Using the data obtained in this study, we developed a model for *iol* gene transcription (Fig. 6). We propose that the heterogeneous phenotype of *S.* Typhimurium growth with MI is essentially related to the mode how IolR interacts with the promoters of those genes that are required for MI utilization. The *iolR* promoter exhibits a non-linear response to growth in MI with two subpopulations of low and high expression level as indicator for bistability^18^. The bistable growth phenotype might be caused by varying copy numbers of heterogeneously transcribed IolR in a sense that IolR dimers do not bind or bind to one or to both of the binding sites, depending on the copy numbers of the repressor. FC analysis demonstrated that during growth with MI, *iolR* expression increases. The IolR molecules, however, are not able to bind *iol* promoters, possibly due to the presence of the catabolic intermediate DKGP that may induce the release of IolR from its target promoters and fine-regulate the repression by IolR. Then, the high amount of inactived IolR permits rapid re-silencing of the *iol* genes upon DKGP dissociation if additional MI is not available or has been replaced by a more optimal carbon and energy source, thus allowing a quick response to changing environments.

The average number of *S*. Typhimurium cells harbouring P_*iolE*_ in the ON status is remarkably low in the absence of MI^4^. Therefore, in light of the high binding affinity of IolR, we speculated that a strong selective force acts on the cells. To test this hypothesis, we used a deletion mutant of *iolR* in which the repression of most *iol* genes is abolished, a situation that at least partially mimics that of cells with an activated *iolE* promoter. In competitive growth experiments with cross-over labelled strains, strain 14028 clearly outcompeted 14028 Δ*iolR* (Fig. 3). The outcome of this experiment led us to conclude that cells that do not use MI but express *iol* genes have a significant fitness disadvantage and are outgrown by cells in which IolR tightly represses expression of these genes. We calculated a high growth rate reduction by ~15% of 14028 *iolR*::Kan^R^. This is in line with the finding that a misfolded YFP protein of resulted in growth disadvantage of 3.2% in yeast^19^, because up to 16 enzymes are untimely produced in a *iolR*-negative strain.

In a cross-over labelled experiment involving kanamycin-resistant strain 14028 *dacB*::Kan^R^ and kanamycin-sensitive strain 14028 ∆*iolR*, we excluded the possibility that reduced fitness is attributable to the antibiotic resistance^20^. Interestingly, when macrophage infection assays were performed^21^, the survival rate of a *iolR* knockout mutant was reduced to only 32% in comparison with the parental strain 14028 (data unpublished). This phenotype may be attributed to the growth disadvantage of the repressor mutant revealed here.

While biological costs of antibiotic resistances or reporter proteins have been studied in detail^22^,^23^, much less reports are available on costly metabolic pathways in case of untimely regulation. Experiments with the lactose operon led the assumption that those costs are associated with transcription and/or translation, but not with the products themselves^24^. More recently, however, this statement was challenged by the hypothesis that the lac permease activity is the main physiological burden to *E. coli*^25^. A recent example of biological costs of gene induction in a pathogen was studied in a fraction of cells harbouring an active type three secretion system (T3SS), which is involved in the virulence of *S*. Typhimurium. The authors reported the retarded growth of cells exhibiting the T3SS-positive phenotype relative to those not expressing the T3SS^26^. A similar observation is the growth restriction of *Yersinia* strains under T3SS-inducing conditions after two generations^27^.

Regulatory mechanisms have probably been evolved to manage tradeoffs for the fitness costs imposed by gene expression and enzymatic activities^28^. We therefore hypothesize here that the strength of the transcriptional repression of the *iol* genes is an evolutionary result of the balance between the expression of a metabolic capability that would be useful in niche occupation, and the fitness cost imposed by such a pathway when an alternative to the respective substrate is present.

## Methods

### Bacterial strains, plasmids and growth conditions

Bacterial strains and plasmids used in this study are listed in Table 2. Strain MvP101 was chosen for safety reasons. Comparison with a shotgun sequence of MvP101 confirmed that its GEI4417/4436 sequence is identical to that of ATCC strain 14028. *S.* Typhimurium strains were grown at 37 °C in Luria-Bertani (LB) medium (10 g/L tryptone, 5 g/L yeast extract and 5 g/L NaCl) or minimal medium (MM). MM comprised M9 medium supplemented with 2 mM MgSO_4_, 0.1 mM CaCl_2_ and 55.5 mM (1%, wt/vol) MI or 25.2 mM (0.5%, wt/vol) glucose. A solution of 1.5% agar (wt/vol) was supplemented to yield solid media. For all FC experiments, bacterial strains were grown overnight at 37 °C in LB medium or MM and subsequently diluted to 1:500 into fresh medium. Bacterial growth curves were obtained from cultures incubated at 37 °C without agitation in 250-ml bulb flasks containing 50 ml of medium. The optical density at 600 nm (OD_600_) was measured at the indicated time points.

### Standard procedures

DNA manipulations and chromosomal or plasmid DNA isolation were performed according to standard protocols^10^ and the relevant manufacturers’ instructions. Plasmid DNA was transformed *via* electroporation using a Bio-Rad Gene Pulser II as recommended by the manufacturer (Bio-Rad, Hercules, CA, USA) and as described previously^21^. Polymerase chain reactions (PCRs) were performed using *Taq* polymerase (Fermentas, St. Leon-Rot, Germany). Chromosomal DNA, plasmid DNA or cells from a single colony were used as a PCR template. *S.* Typhimurium 14028 gene numbers refer to the LT2 annotation (NC 003197). Statistical analyses were performed with ANOVA or t-test; three levels of significance were considered (p < 0.05, p < 0.01 and p < 0.001).

### Cloning of *gfp* reporter strains

The *rpsM* and *iolR* promoters defined as the 500 bp upstream of the start codons, and the last 500 bp of the *iolR* coding sequence were amplified from the chromosomal DNA of strain 14028 by PCR using the oligonucleotides listed in Table S1. The fragments were cloned *via* KpnI (Fermentas) upstream of *gfp* into the multiple cloning site of the suicide vector pUTs-*gfp*(Cm^R^) and amplified in *E. coli* S17.1. Plasmids containing the correct *gfp*-fusions were isolated, verified by PCR and transferred into *S.* Typhimurium 14028 via conjugation. Strains with chromosomal insertions were selected, and validated by DNA sequencing (GATC, Konstanz, Germany).

### Generation of an *iolR* deletion mutant

The one-step method based on phage λ Red recombinase cassette^2^,^29^ was used for the allelic replacement of *iolR* by a kanamycin-resistance cassette (Kan^R^). To exclude unspecific recombination events, 5 ml (OD_600_) of 14028 donor cells harbouring the Kan^R^ were mixed with 5 μl of P22 phage suspension and incubated for 6 h without agitation. The culture was stored for 2 h at 4 °C, after which the cells were sedimented via centrifugation at 7000 rpm and 4 °C for 10 min. The supernatant was filter sterilized through a 0.2-μm pore filter and stored at 4 °C. Recipient MvP101 cells were prepared from an overnight culture, mixed at a 1:20 ratio with P22 lysate and incubated for 1 h at 37 °C without agitation. After incubation, the mixture was plated on selective agar-plates and incubated at 37 °C. To obtain phage-free colonies, bacteria were cultivated on green indicator plates^30^. A nonpolar deletion mutant was then obtained upon transformation with pCP20. Gene deletions were verified by PCR analysis and DNA sequencing.

### Flow cytometry (FC)

Single cell analysis was performed on a FACSAria II analyzer (Becton Dickinson, San Jose, CA, USA) at an excitation wavelength of 488 nm. Light emission from green fluorescent protein (GFP) was measured between 515 and 545 nm. For all assays, independent overnight cultures of *S.* Typhimurium strain MvP101 carrying a chromosomal fusion of *gfp* with P_*iolR*_, *iolR* or the promoter of the house-keeping gene *rpsM* (P_*rpsM*_) were diluted 1:500 into 50 ml of MM with MI or glucose and incubated in 250-ml bulb flasks. Samples were collected at the indicated time points and diluted in 1% phosphate-buffered saline (PBS) containing 2% formaldehyde to ensure GFP stability^31^. For all FC experiments, 10,000 events were recorded and the collected data were analyzed using Flowing Software (v 2.5.1; http://www.uskonaskel.fi/flowingsoftware/). To exclude doublets, we applied the *Area Scaling* function of the FACS Aria II that adjusts the pulse area with the pulse level of the *forward scatter* signal. Thus, cells of the same size exhibit a similar pulse area and level, and can be identified in a scatter diagram. To separate the single cells from the doublets, we used a gate on the diagonal of the diagram.

### Overproduction and purification of IolR

IolR-His_6_ was overproduced from the plasmid pET28b-IolR in *E. coli* BL21 (DE3) and *S.* Typhimurium strain 14028^2^. Overnight culture of the strains were diluted 1:100 into 400 ml of LB medium supplemented with 50 μg/ml kanamycin and incubated for 3 h at 37 °C with rotation at 180 rpm. At an OD_600_ of 0.6, IolR production was induced by adding 0.1 mM isopropyl-β-_D_-1-thiogalactopyranoside (IPTG). After an overnight incubation at 37 °C and 180 rpm, the cells were harvested by centrifugation at 4 °C and 7500 rpm for 20 min. The pellets were each resuspended in 5 ml of native lysis buffer (50 mM NaH_2_PO_4_, 300 mM NaCl and 10 mM imidazole at pH 8.0) and lysed by three passages through a French press (SLM Aminca Instruments, Rochester, NY, USA) at 900 psi; residual cell debris was removed twice by centrifugation at 4 °C and 9000 rpm for 15 min. Following filtration, IolR was isolated using a HisTrap HP colunm with an ÄKTA purifier 10 system (GE Healthcare, Little Chalfont, UK). The protein concentration was determined using RotiQuant solution (Carl, Roth GmbH, Karlsruhe, Germany) according to the Bradford method^32^. The purity of the eluted fractions was analysed by separation on a 15% sodium dodecyl sulfate (SDS)-PAA gel.

### Electrophoretic mobility shift assays

Fragments of promotor regions were amplified (for oligonucleotides, see Table S1) as decribed above, and 100 ng of DNA were mixed with increasing amounts of purified IolR-His_6_ in 1× Tris/borate/EDTA (EMSA) buffer to a total volume of 20 μl. After incubation for 45 min, the samples were mixed with 4 μl of 6× loading dye (MBI Fermentas) and loaded on a 12% native PAA gel prepared in EMSA buffer; the samples were then separated at 120 V and 4 °C for 2 h in the same buffer. Gel-bound DNA was stained using GelRed (Biotium, Hayward, CA, USA) and visualized by ultraviolet (UV) irradiation.

### Biotinylated double-stranded (BIOT-ds) DNA fragments

BIOT-ds DNA fragments were obtained by annealing ss-DNA-oligonucleotides or via PCR. BIOT-P_*iolT1*_, BIOT-P_*iolR*_ and BIOT-P_*reiD*_ were amplified by PCR using the respective biotinylated DNA oligonucleotides (Sigma-Aldrich, Deisenhofen, Germany) and chromosomal DNA from strain 14028 as a template. To assemble the control promoter region P_*pcfA*_ from *Photorhabdus luminescens*^33^, oligonucleotides P4568-btn_fw and P4568_rew were incubated for 5 min at 100 °C, mixed and cooled prior to annealing. BIOT-ds DNA fragments were then captured on SPR sensor chips.

### SPR spectroscopy and calibration-free concentration analysis (CFCA)

SPR spectroscopy and CFCA assays were performed using a Biacore T200 device (GE Healthcare) and streptavidin-precoated Xantec SAD500-L carboxymethyl dextran sensor chips (XanTec Bioanalytics GmbH, Düsseldorf, Germany). All experiments were conducted at 25 °C with HBS-EP+ buffer [10 mM HEPES pH 7.4, 150 mM NaCl, 3 mM EDTA and 0.05% (v/v) detergent P20] as previously described in detail^17^.

Assuming a globular shape, the diffusion coefficient of IolR was calculated using the Biacore diffusion constant calculator and converter webtool (https://www.biacore.com). The diffusion coefficient of IolR was determined to be D = 9.946 × 10^−11^ m^2^/s. The initial rates of the dilutions that differed by a factor of at least 1.5 were considered when calculating the concentration of IolR that interacted with the ligand. This “active” protein concentration, which was determined as 3.3 × 10^−8^ M (66% of the total protein concentration), was then used to calculate the binding kinetic constants and steady-state affinity.

### Interaction Map (IM) analysis

IM analyses were performed as previously described^17^,^34^. SPR spectroscopy curves were determined as the sums of individual binding curves, each of which represented a monovalent interaction^35^ with a unique combination of the association rate *k*_*a*_ (ON-rate) and dissociation rate *k*_*d*_ (OFF-rate) and the consequently unique real equilibrium dissociation constant *K*_*D*_ = *k*_*d*_/*k*_*a*_. This algorithm was used to split the experimental SPR spectroscopy data set into several theoretical monovalent binding curves and the binding curves that, when summed, best fit the experimental data. The association rate *k*_*a*_ and dissociation rate *k*_*d*_ were plotted within a two-dimensional distribution yield a heterogeneous binding data display in the form of a map wherein each peak corresponded to a single component that contributed to the cumulative binding curve^36^. To further quantify protein/DNA interactions, the OneToTwo evaluation algorithm, which is available in the Trace Drawer Software package (Ridgeview Diagnostics AB, Uppsala, Sweden), was used.

## Additional Information

**How to cite this article:** Hellinckx, J. *et al*. High binding affinity of repressor IolR avoids costs of untimely induction of *myo*-inositol utilization by *Salmonella* Typhimurium. *Sci. Rep.* **7**, 44362; doi: 10.1038/srep44362 (2017).

**Publisher's note:** Springer Nature remains neutral with regard to jurisdictional claims in published maps and institutional affiliations.

## Supplementary Material

Supplementary Information

## Figures and Tables

**Table 1:** Fluorescence variance (noise level) of MvP101 P_*iolR*_::*gfp* and MvP101 *iolR*::*gfp* from three independent growth experiments in MM/MI.

	Hours	noise-level with s.d.	growth phase
MvP101 P_*iolR*_::*gfp*	0	1.41 ± 1.05	lag phase
	4	0.45 ± 0.02	
	10	0.16 ± 0.01	
	14	0.06 ± 0.02	log phase
	18	0.19 ± 0.06	
	38	0.12 ± 0.00	stationary phase
	48	0.08 ± 0.00	
MvP101 *iolR*::*gfp*	0	1.67 ± 0.13	lag phase
	24	1.68 ± 0.58	
	26	1.49 ± 0.95	
	28	0.41 ± 0.29	log phase
	30	0.17 ± 0.10	
	32	0.12 ± 0.07	
	34	0.13 ± 0.08	

s. d., standard deviation.

**Table 2:** Strains and plasmids used in this study.

Bacterial strains	Description and relevant features	Source or literature
14028	*S.* Typhimurium strain ATCC14028	ATCC
14028 *iolR*::Kan^R^	Allelic-exchange mutant	This study
14028 ∆*iolR*	In-frame *iolR* (STM4417) deletion mutant	This study
14028 *dacB*::Kan^R^	Allelic-exchange mutant	This study
14028 *iolR*::Kan^R^ ∆4418–4436	Deletion of *iolT1* (STM4418) to *iolH* (STM4436) in 14028 *iolR*::Kan^R^	This study
MvP101	14028 with *sseD*::*aphT*, Kan^R^; allelic-exchange mutant	37
MvP101 ∆*iolR*	In-frame *iolR* (STM4417) deletion mutant of MvP101	This study
*E. coli* BL21(DE3)	F^−^, *ompT, hsd*S_B_ (r_B_^−^m_B_^−^), *gal, lon, dcm, rne*131, λ (DE3 [*lacI lac*UV5-T7 gene 1 *ind*1 *sam*7 *nin*5]	38
Plasmids		
pKD3	*pir*-dependent, FRT sites, Cm^R^	29
pKD4	*pir*-dependent, FRT sites, Kan^R^	29
pKD46	Lambda-Red helper plasmid; Amp^R^	29
pCP20	FLP recombinase plasmid; Amp^R^	29
pUTs-*gfp*(Cm^R^)	Replacement of *lux* with *gfp* from pPROBE-NT in a transposase-negatvive derivate of pUT mini-Tn5 luxCDABE Km2; suicide plasmid, *mob*RP4, *ori* R6K, *gfp*, Cm^R^	39
pUTs-P_*iolR*_::*gfp*	pUTs-*gfp*(Cm^R^) with the 500 bp region upstream of *iolR* (transcriptional fusion to *gfp*)	This study
pUTs-*iolR*::*gfp*	pUTs-*gfp*(Cm^R^) with the last 500 bp of *iolR* (translational fusion to *gfp*)	This study
pUTs-P_*rpsM*_::*gfp*	pUTs-*gfp*(Cm^R^) with the 500 bp region upstream of *rpsM* (STM3418) in front of *gfp*	This study
pBR-*iolR*	*iolR* with promoter region cloned into pBR322	2
pET28b-*iolR*	Expression vector, T7*lac* promoter; Kan^R^	2
