# Supplementary material for: High binding affinity of repressor IolR avoids costs of untimely induction of myo-inositol utilization by Salmonella Typhimurium
Source: Sci Rep. 2017 Mar 14;7:44362. doi: 10.1038/srep44362 (PMC5349611; doi:10.1038/srep44362)
Supplement: Supplementary Information [file srep44362-s1.docx]

**Supplementary Information**

**High binding affinity of repressor IolR avoids costs of untimely induction of *myo*-inositol utilization by *Salmonella* Typhimurium**

Jessica Hellinckx^1^, Ralf Heermann^2^, Angela Felsl^1^ and Thilo M. Fuchs^1,3^*

^1^Lehrstuhl für Mikrobielle Ökologie, Zentralinstitut für Ernährungs- und Lebensmittelforschung ZIEL, Technische Universität München, Weihenstephaner Berg 3, 85354 Freising, Germany; ^2^Biozentrum, Bereich Mikrobiologie, Ludwig-Maximilians-Universität München, Großhaderner Str. 2-4, 82152 Martinsried/München, Germany; ^3^Friedrich-Loeffler-Institut, Institut für Molekulare Pathogenese, Naumburger Str. 96a, 07743 Jena, Germany

**Figure S1.** **Growth curves of 14028, 14028 ∆*iolR*, 14028 *dacB*::Kan^R^ and 14028 *iolR*::Kan^R^ in LB medium.** An overnight culture was adjusted to an OD_600_ of 0.8 and diluted 1:1000 into fresh LB medium. Growth behaviour was monitored in microtiter plates incubated at 37°C and 500 rpm. Standard deviations were calculated from three independent overnight cultures and three replicates.





**Table S1.** Oligonucleotides used in this study.

| primer name | target gene | modification | 5‘ → 3‘ sequence |
| --- | --- | --- | --- |
| *construction of pUTs suicide vectors* | | | |
| PiolR_KpnI_for | *STM4417 (iolR)* | *Kpn*I | CGGggtaccCAGCGTATTAAATGAC |
| pUTs4417up rev KpnI |  | *Kpn*I | CGGggtaccGCATGATACTCTAATAG |
| iolR_KpnI_for | *STM4417 (iolR)* | *Kpn*I | CGGggtaccAAAATGCGGATGAAGGGG |
| iolR_KpnI_rev |  | *Kpn*I | CGGggtaccCCAGTGAAACCGCTAACG |
| PrpsM_KpnI_for | *STM3418 (rpsM)* | *Kpn*I | CGGggtaccCCGCGTGAAACAGC |
| PrpsM_KpnI_rev |  | *Kpn*I | CGGggtaccGCCAGCTCAACCCA |
| *construction of non-polar deletion mutants** | | | |
| 5del_4417for | *STM4417 (iolR)* |  | TACGAAATTTTCGTTCTATTAGAGTATCATGCATGTCTAAACATCAAACTGTGTAGGCTGGAGCTGCTT |
| 3del_4417rev |  |  | ATCGGCTTGTTTTTTTACTCCGTCGCCAGCGCCAGTGAAACCGCTAACGTCATATGAATATCCTCCTTA |
| dacB_fwd_Del | *STM3300 (dacB)* |  | ACGGCTTGTTTCCAGGGTGTTAGCGCGAGATTATGCGATTTTCCAGATTTGTGTAGGCTGGAGCTGCTTC |
| dacB_rev_Del |  |  | CCGGCAATAAATGCACTCAGTTATTCTGATAAATATCCTTATACAACCGACTCCATATGAATATCCTCCTTA |
| *test of insertion of kanR and gene deletion*** | | | |
| test4417_5'for | *STM4417 (iolR)* |  | TATGTTCAGTTCATTTGTGC |
| test4417_3'rev |  |  | TATTCTGATTAAGTTTCACC |
| dacB_fwd | *STM3300 (dacB)* |  | GGGCAATGAACTCTACCC |
| dacB_rev |  |  | GCAGCTGGCTGAAGTGGAAG |
| kanR1 | *kanR* |  | GTATGCAGCCGCCGC |
| kanR4 |  |  | CGATGCCTGCTTGCCG |

| primer name | target gene | modification | 5‘ → 3‘ sequence |
| --- | --- | --- | --- |
| *cloning, testing and sequencing of reporter fusions with pUTs-gfp*** | | | |
| GFPtestR | *gfp* |  | CCTTCACCCTCTCCAC |
| pUTs_PiolR_fo | *STM4417 (iolR)* |  | TGCGGATGAAGTGTTCC |
| PiolR_KpnI_for | *STM4417 (iolR)* | *Kpn*I | CGGggtaccCAGCGTATTAAATGAC |
| PrpsM_testF | *STM3418 (rpsM)* |  | GTGGTCAACGCCGTAT |
| *constructions of fragments used in EMSA assays*** | | | |
| PiolR_1.1F | *STM4417 (iolR)* |  | TTGCTCAGAACCCAGC |
| PiolR_1.1R |  |  | CTGCGGATATCATCCTGC |
| PiolR_1.3F |  |  | CACTAAGAGTGTTCCGCC |
| PiolR_1.2R |  |  | GTACAGAATTTGCTGGCG |
| PiolR_1.3R |  |  | CGCAAACGTTGAAAGGC |
| PiolR_1.2F |  |  | GCCTTTCAACGTTTGCG |
| PiolT1_1.1R | *STM4418 (iolT1)* |  | CAATCGCGCAGATGC |
| PiolT1_1.1F |  |  | GCTACGGCAATGACCG |
| PiolT1_1.2R |  |  | CTTATGTCGGTCATTGCC |
| PiolT1_1.2F |  |  | AGCACTGCGTAAAACG |
| PiolT1_1.3R |  |  | TTTACGCAGTGCTCTCC |
| PiolT1_1.3F |  |  | CGAAATGGAGATGTTCCAGG |
| PiolT1_1.4F |  |  | AGTTCAGGTACTGTCGCG |
| PiolT2_1.1F | *STM4419 (iolT2)* |  | AATTTGTCAGGGGCCG |
| PiolT2_1.1R |  |  | ACGTACACATAGGCCG |
| PiolC1_1.1R | *STM4430 (iolC1)* |  | TCCAGCGGCTTTTGC |

| primer name | target gene | modification | 5‘ → 3‘ sequence |
| --- | --- | --- | --- |
| PiolC1_1.1F |  |  | ATCATGCCCGTTGCC |
| PiolC1_1.2R |  |  | CATTTCCGGGCAACG |
| PiolC1_1.2F |  |  | TCGACATTCAGGCG |
| PiolC1_1.3R |  |  | ACGCCTGAATGTCG |
| PiolC1_1.3F | *STM4430 (iolC1)* |  | ATGCATCGCTTCAGCC |
| PiolC1_1.4F |  |  | ATGGGAAGGAGATC |
| PiolD1_1.1F | *STM4431 (iolD1)* |  | CGATGCATAAGCAGGC |
| PiolD1_1.1R |  |  | AAAGCGCACCAAAGCC |
| PiolA_1.1F | *STM4421 (iolA)* |  | CTGCTGCTCAGTGTCG |
| PiolA_1.1R |  |  | GCTGCACATACCCTCG |
| PiolA_1.2F | *STM4423 (reiD)* |  | CGAGGGTATGTGCAGC |
| PiolA_1.2R |  |  | TGTGGTGCCGAAACCC |
| PiolA_1.3F |  |  | TTCAGGGGGGTTTCGG |
| PiolA_1.3R |  |  | AACTGGGGCTGAGC |
| PiolA_1.4R |  |  | CCATTGATGACACACC |
| PiolA_1.5F |  |  | GGTGTGTCATCAATGG |
| PiolA_1.4R |  |  | CCATTGATGACACACC |
| PiolA_1.5F |  |  | GGTGTGTCATCAATGG |
| PreiD1F |  |  | GGTTGTCTGTTGTGACGAGG |
| PreiD1R |  |  | CCACACCAAATGAAACAAAAATGC |
| PreiD2F |  |  | GGTGTGGTATAAAAATTCAAATTTCAGG |
| PreiD2R |  |  | GCACAAGCGCATGATTTACC |

| primer name | target gene | modification | 5‘ → 3‘ sequence |
| --- | --- | --- | --- |
| primer name | target gene | modification | 5‘ → 3‘ sequence |
| *fragments used for Surface Plasmon Resonance Spectroscopy* | | | |
| PiolR_SPR_F | *STM4417 (iolR)* | *5’ biotinylated* | TTGCTCAGAACCCAGC |
| PiolR_SPR_R |  | *5’ biotinylated* | CTGCGGATATCATCCTGC |
| PiolT1_SPR_F | *STM4418 (iolT1)* | *5’ biotinylated* | AACGATAAAAAACGCCAG |
| PiolT1_SPR_R |  | *5’ biotinylated* | CTTATGTCGGTCATTGCC |
| PreiD_SPR_F | *STM4423 (reiD)* | *5’ biotinylated* | GGTGTGTCATCAATGG |
| PreiD_SPR_R |  | *5’ biotinylated* | TGTGGTGCCGAAACCC |
